# Editorial for Special Issue on Biomimetic Adaptive Buildings

**DOI:** 10.3390/biomimetics10120844

**Published:** 2025-12-17

**Authors:** Negin Imani, Brenda Vale, Derek Clements-Croome

**Affiliations:** 1 Bodeker Scientific, Wellington 6012, New Zealand; 2 Centre for Sustainability, University of Otago, Dunedin 9016, New Zealand; 3 Open Polytechnic, Lower Hutt 5011, New Zealand; 4 Wellington Faculty of Architecture and Design Innovation, Victoria University of Wellington, Wellington 6140, New Zealand; brenda.vale@vuw.ac.nz; 5 School of the Built Environment, University of Reading, Reading RG6 6UR, UK; d.j.clements-croome@reading.ac.uk

It seems that the future of building envelopes is moving towards adaptivity and self-regulation, reflecting the growing view that a vital strategy in addressing climate change is understanding buildings as living systems rather than static entities. This shift in perspective encourages the development of façades capable of dynamically responding to environmental conditions. Such systems can autonomously regulate temperature, daylight, and airflow, thereby reducing operational energy demand while enhancing indoor comfort.

Such a reduction is important, as buildings account for 30% of global energy use and 27% of emissions [1]. Biomimetic Adaptive Buildings (Bio-ABFs) can reduce building energy use and enhance thermal comfort [2,3,4,5,6,7,8,9] by 50% [10] and 37.1%, respectively [11]. Living building technologies rely on active or passive self-regulating mechanisms. Active systems utilise external components, such as sensors, processors, and actuators, to achieve adaptive behaviour. Recent innovations include self-adjusting shading systems, dynamic insulation, and responsive ventilation technologies that enable real-time, automated performance control. In contrast, passive systems rely on intrinsic material properties and structural characteristics that respond naturally to changing environmental conditions. As highlighted by the International Energy Agency’s 2022 report [12], conventional materials with static behaviour fall short in response to changing climatic conditions.

As technology rapidly develops, contemporary buildings face increasingly complex environmental challenges. Architects and engineers are therefore called to rethink the relationship between the built environment and its surroundings. Conventional, static design approaches are often insufficient in effectively responding to fluctuating climatic conditions, resource limitations, and evolving occupant comfort and wellbeing standards. This Special Issue, “Biomimetic Adaptive Buildings,” examines an emerging paradigm in which buildings emulate nature’s capacity for adaptation, efficiency, and resilience. As most buildings last for decades, if not more, such an adaptation is crucial.

Biomimetic design has long inspired architecture, from the thermoregulatory efficiency of termite mounds to the structural ingenuity of plant stems. Recent advances in smart materials, sensor systems, and computational design now allow these biological principles to be implemented as functional architectural technologies. This Special Issue presents a range of contributions exploring how natural intelligence can inform the material, systemic, and ecological adaptability of built environments.

Our Special Issue brings together seven original research articles and one review paper that collectively explore the breadth of biomimetic adaptive building design. The contributions cover diverse aspects, including adaptive façades, bio-based materials, energy generation, environmental performance, and integrated design assessment frameworks.

A principal focus of this issue is adaptive façade systems, building envelopes capable of dynamically responding to environmental stimuli such as temperature, humidity, and solar radiation. Imani et al. [13], in “Biomimetic Shading Systems: Integrating Motorised and Moisture-Responsive Actuation for Adaptive Façades,” present a two-stage investigation combining mechanical and passive adaptation. Nine motorised shading concepts inspired by plant stomata thermoregulation are developed, followed by a passive actuation prototype employing a polylactic acid (PLA) biocomposite and regenerated cellulose fibres. The findings indicate that such materials can autonomously alter geometry in response to humidity, offering a low-energy pathway toward climate-responsive façades.

In [14], “Biomimetic Adaptive Building Façade Modelling for Sustainable Urban Freshwater Ecosystems,” Kahvecioğlu et al. [14] propose a sun-shading façade system inspired by lizard skin microstructures and leaf venation. The façade collects and channels rainwater to support urban freshwater ecosystems, extending its functionality beyond thermal regulation to an integrated ecological role. Similarly, Valinejadshoubi, Athienitis [15] present a dynamic photovoltaic façade combining shading control with energy generation, demonstrating significant improvements in thermal and visual comfort while reducing overall energy demand. The façade’s responsiveness to changing solar conditions exemplifies how building envelopes can actively engage with their environment rather than remaining static barriers.

Sankaewthong et al. [16] investigate the Mimosa kinetic façade, inspired by the plant’s adaptive movements, as a dynamic solution to improve indoor air quality and occupant comfort in densely populated buildings. Tests in Bangkok revealed that specific façade patterns excelled in terms of ventilation, while others optimised long-term comfort. Double-sided designs enhanced cross-ventilation and air purification, achieving airflow of up to 12 m/s compared to the 2.5 m/s seen with static façades. These findings highlight the potential of kinetic systems in transforming sustainable building practices; however, further research is needed to validate performance across diverse climates and real-world conditions.

AlAli et al. [17] explore nature-inspired design in laboratory buildings, emphasising biomimicry and biophilic principles to enhance sustainability and human well-being. They introduce the Nature-Inspired & Living Laboratory (NILL 1.0)™ Building Assessment Index, a framework for evaluating how buildings integrate sustainable practices, regenerative features, and occupant-focused design. By linking building systems to natural and human body analogies, the index guides the creation of laboratories that are resilient, environmentally responsible, and supportive of scientific innovation. Collectively, these studies illustrate how façades can evolve from static barriers into multifunctional, responsive, and ecologically engaged building skins.

The second thematic cluster bridges adaptive building concepts and sustainable, bio-based materials. Nicolalde et al. [18] evaluate two Ecuadorian woods—Guayacan and oaks—as sustainable construction alternatives. Results show that Guayacan has lower mechanical strength and thermal conductivity, while oak properties vary by region; more specifically, Manabi oak exhibits slightly better thermal performance, while Loja oak shows superior elasticity. Multicriteria decision methods, such as the entropy-COPRAS approach, provide an objective framework for selecting timber based on varying mechanical and thermal properties, supporting informed building material choices.

El Haddaji et al. [19] explore Brewers’ Spent Grains (BSGs) combined with cornstarch as a sustainable biocomposite, comparing the mixture with conventional hemp shives. While the BSG formulation exhibits higher thermal conductivity, it demonstrates superior hygrothermal performance, and its mechanical properties are comparable across all formulations. These findings indicate BSG waste as a promising construction material that supports circular economy principles; however, further research is needed to optimise formulations and assess durability.

Complementing the original research articles, this volume also includes a comprehensive review paper examining the role of biomimicry in architecture for improving energy efficiency and building performance. This review highlights how natural strategies, particularly those found in plants, can inspire adaptive and sustainable design solutions. It presents a synthesis of global case studies, demonstrating the versatility and effectiveness of biomimetic approaches across diverse climatic and architectural contexts [20].

Across these thematic strands, the contributions of this Special Issue collectively redefine building adaptation. From moisture-responsive shading modules and waste-derived composites to sensor-driven façades, adaptive design requires the integration of material behaviour, technological intelligence, and ecological connectivity. This collection aims to serve as a reference for future interdisciplinary research, fostering collaboration among architects, engineers, material scientists, and biologists. As biomimetic thinking continues to evolve, the ultimate objective is not merely to replicate natural forms but to embed processes of adaptation and regeneration into the built environment itself.

The guest editors extend their sincere gratitude to all contributing authors for their innovative research, to the reviewers for their thoughtful evaluations, and to the *Biomimetics* (MDPI) editorial team for their support and professionalism. Recognition is also given to the academic institutions, funding organisations and research centres advancing biomimetic design through experimentation, teaching, and cross-disciplinary collaboration.
